# Virtual self-conversation to support people living with obesity when starting their change process towards a healthier lifestyle: Preliminary results of a longitudinal study

**DOI:** 10.1192/j.eurpsy.2023.302

**Published:** 2023-07-19

**Authors:** P. Lusilla, D. Anastasiadou, P. Herrero, J. Vazquez, B. Spanlang, M. Slater, J. A. Ramos, G. Parramon, A. Ciudin, M. Comas

**Affiliations:** 1 Psychiatry, Huvh; 2 Psychiatry, Vhir; 3 Virtual BodyWorks; 4 Endocrinology; 5 Obesity Unit, Huvh, Barcelona, Spain

## Abstract

**Introduction:**

People living with obesity (PLWO) often experience ambivalence when starting their change process towards a healthier lifestyle. Psychological treatments for obesity should resolve this ambivalence and help PLWO to explore their own reasons for change in line with their needs and values, as well as promote self-efficacy. Following the Motivational Interviewing (MI) principles, the SOCRATES project proposes a “virtual self-conversation” to help PLWO to address some of the psychological aspects associated with obesity, such as the lack of awareness about their condition, the impact of the internalization of weight stigma, and the lack of self-efficacy.

**Objectives:**

With the current longitudinal study, we aim to explore how the participants’ process of lifestyle change, and how their eating habits and dysfunctional eating patterns change before and after the virtual intervention.

**Methods:**

Forty-eight patients with obesity from the Vall d’Hebron University Hospital (Mean age = 19.7 years) were assigned to 3 groups. The Experimental Group 1 (EG1) (N = 21), after completing an intensive training on MI, received a virtual intervention using the “motivational self-conversation” technique. The Experimental Group 2 (EG2) (N = 17) underwent a virtual intervention with a pre-registered psychoeducational dialogue, and the Control Group (CG) (N = 10) followed treatment-as-usual. All participants completed self-reported questionnaires on their motivation to change lifestyle [(*Readiness Rulers (RR), Processes of Change questionnaire in weight management (P-W)*], eating habits (*Habits questionnaire*) and dysfunctional eating patterns (*Three Factor Eating Questionnaire-18*) at baseline (T0), post-intervention (T1), and 4 weeks follow-up (T2). Repeated measures ANOVA was performed for all the questionnaires.

**Results:**

Statistically significant results were shown regarding motivation to change through the RR and the “evaluation of the consequences of their weight” subscale of P-W across time for the EG1 (p < .05). These results suggest that participants’ motivation to eat healthier and do more exercise, as well as self-awareness about the negative consequences of their condition increased after the virtual intervention.

**Conclusions:**

The present study showed that this novel virtual intervention might be an effective tool in helping PLWO resolve their ambivalence to change lifestyle and acquire self-awareness about their condition. However, the intervention did not lead to significant changes in other psychological variables, such as lifestyle habits or dysfunctional eating patterns; domains that may be less sensitive to changes over the time, and which may take place once motivation is well-established.

**Disclosure of Interest:**

None Declared

